# Liver trauma: WSES 2020 guidelines

**DOI:** 10.1186/s13017-020-00302-7

**Published:** 2020-03-30

**Authors:** Federico Coccolini, Raul Coimbra, Carlos Ordonez, Yoram Kluger, Felipe Vega, Ernest E. Moore, Walt Biffl, Andrew Peitzman, Tal Horer, Fikri M. Abu-Zidan, Massimo Sartelli, Gustavo P. Fraga, Enrico Cicuttin, Luca Ansaloni, Michael W. Parra, Mauricio Millán, Nicola DeAngelis, Kenji Inaba, George Velmahos, Ron Maier, Vladimir Khokha, Boris Sakakushev, Goran Augustin, Salomone di Saverio, Emanuil Pikoulis, Mircea Chirica, Viktor Reva, Ari Leppaniemi, Vassil Manchev, Massimo Chiarugi, Dimitrios Damaskos, Dieter Weber, Neil Parry, Zaza Demetrashvili, Ian Civil, Lena Napolitano, Davide Corbella, Fausto Catena, the WSES expert panel, Hany Bahouth, Matti Tolonen, Paola Fugazzola, Jose Julian Serna, Fernando Rodriguez, Alberto F. García, Adolfo Gonzalez, Luis Fernando Pino, Mónica Guzmán-Rodríguez, Bruno M. Pereira, Andrew Kirkpatrick, Alain Chichom Mefire, Antonio Tarasconi, Osvaldo Chiara, Carlos Augusto Gomes, Joseph Galante, Miklosh Bala, Paola Perfetti, Fernando Machado, Oreste Romeo, Francesco Salvetti, Lorenzo Ghiadoni, Francesco Forfori, Paolo Malacarne, Silvia Pini, Marsia Pucciarelli, Marco Ceresoli, Catherine Arvieux, Denis Khokha, David A. Spain, Arda Isik

**Affiliations:** 1 General, Emergency and Trauma Surgery Department, Pisa University Hospital, Via Paradisia 1, 56100 Pisa, Italy; 2 Riverside University Health System, CECORC Research Center, Loma Linda University, Loma Linda, USA; 3 Division of Trauma and Acute Care Surgery, Fundación Valle del Lili, Cali, Colombia; 4 Division of General Surgery, Rambam Health Care Campus Haifa, Haifa, Israel; 5 Department of Surgery, Hospital Angeles Lomas, Huixquilucan, Mexico; 6 Trauma Surgery, Denver Health, Denver, CO USA; 7 Trauma Surgery Department, Scripps Memorial Hospital La Jolla, San Diego, CA USA; 8 Surgery Department, University of Pittsburgh, Pittsburgh, PA USA; 9 Department of Cardiothoracic and Vascular Surgery, Örebro University Hospital, Örebro University, Örebro, Sweden; 10 Department of Surgery, College of Medicine and Health Sciences, UAE University, Al-Ain, United Arab Emirates; 11 General and Emergency Surgery, Macerata Hospital, Macerata, Italy; 12 Trauma/Acute Care Surgery & Surgical Critical Care, University of Campinas, Campinas, Brazil; 13 General, Emergency and Trauma Surgery Department, Bufalini Hospital, Cesena, Italy; 14 Department of Trauma Critical Care, Broward General Level I Trauma Center, Fort Lauderdale, FL USA; 15 Unit of Digestive Surgery, HPB Surgery and Liver Transplant, Henri Mondor Hospital, Créteil, France; 16 General and Trauma Surgery, LAC+USC Medical Center, Los Angeles, CA USA; 17 General and Emergency Surgery, Massachusetts General Hospital, Boston, MA USA; 18 Department of Surgery, Harborview Medical Centre, Seattle, USA; 19 General Surgery Department, Mozir City Hospital, Mozir, Belarus; 20 General Surgery Department, Medical University, University Hospital St George, Plovdiv, Bulgaria; 21 Department of Surgery, Zagreb University Hospital Centre and School of Medicine, University of Zagreb, Zagreb, Croatia; 22 General and Trauma Surgery Addenbrooke’s Hospital, Cambridge University Hospitals NHS Foundation Trust, Cambridge, UK; 23 3rd Department of Surgery, Attiko Hospital, National & Kapodistrian University of Athens, Athens, Greece; 24 Chirurgie Digestive, CHUGA-CHU Grenoble Alpes, Grenoble, France; 25 General and Emergency Surgery, Sergei Kirov Military Academy, Saint Petersburg, Russia; 26 General Surgery Department, Mehilati Hospital, Helsinki, Finland; 27 General and Trauma Surgery Department, Pietermaritzburg Hospital, Pietermaritzburg, South Africa; 28 General and Emergency Surgery, NHS Lothian, Edinburgh, UK; 29 Department of General Surgery, Royal Perth Hospital, Perth, Australia; 30 General and Trauma Surgery Department, London Health Sciences Centre, Victoria Hospital, London, ON Canada; 31 General Surgery, Tbilisi State Medical University, Tbilisi, Georgia; 32 Trauma Surgery, Auckland University Hospital, Auckland, New Zealand; 33 Division of Acute Care Surgery, University of Michigan Health System, Ann Arbor, MI USA; 34 ICU Department, Papa Giovanni XXII Hospital, Bergamo, Italy; 35 Emergency and Trauma Surgery, Maggiore Hospital, Parma, Italy; 36 Department of Surgery, Örebro University Hospital, Örebro University, Örebro, Sweden

**Keywords:** Liver trauma, Adult, Pediatric, Minor, Moderate, Severe, Classification, Guidelines, Surgery, Hemorrhage, Operative management, Non-operative management, Interventional, Radiology, Intensive care

## Abstract

Liver injuries represent one of the most frequent life-threatening injuries in trauma patients. In determining the optimal management strategy, the anatomic injury, the hemodynamic status, and the associated injuries should be taken into consideration. Liver trauma approach may require non-operative or operative management with the intent to restore the homeostasis and the normal physiology. The management of liver trauma should be multidisciplinary including trauma surgeons, interventional radiologists, and emergency and ICU physicians. The aim of this paper is to present the World Society of Emergency Surgery (WSES) liver trauma management guidelines.

## Background

Liver trauma is one of the most common abdominal lesions in severely injured trauma patients [1]. Diagnosis and treatment of hepatic trauma has evolved with the use of modern diagnostic and therapeutic tools [2–4]. Until two to three decades ago, most cases with blunt abdominal trauma and possible injury in parenchymatous organs were managed by exploratory laparotomy [5]. Several innovative multimodal approaches as EVTM (endovascular trauma and bleeding management) have allowed to greatly increase the likelihood of non-operative management (NOM) for selected patients. Nowadays, even borderline patients or transient responder, without other indications for laparotomy, may be considered for NOM in selected and well-developed trauma centers. This advanced strategy necessitates a multidisciplinary approach to deal with the complexity of moderate and severe liver injury. The majority of patients admitted with liver injuries have minor or moderate injuries (WSES I, II, III) (AAST-OIS I, II, or III) and are successfully treated by NOM. In contrast, one third of severe injuries (WSES IV, V) (AAST-OIS IV, V) allow for NOM [6]. In pediatric patients, NOM should be considered the optimal management approach. In determining the optimal treatment strategy, the anatomical description of liver lesions is fundamental but not sufficient. In fact, the decision whether patients need to be managed operatively or undergo NOM is based mainly on the hemodynamic status, associated injuries, and on the anatomical liver injury grade.

The aim of this manuscript is to present the updated World Society of Emergency Surgery (WSES) liver trauma management guidelines.

## Notes on the use of the guidelines

The guidelines are evidence-based, with the grade of recommendation based on the evidence. The guidelines present the diagnostic and therapeutic methods for optimal management of liver trauma. The practice guidelines promulgated in this work do not represent a standard of practice. These are suggested plans of care, based on best available evidence and the consensus of experts, but they do not exclude other approaches as being within the standard of practice. For example, they should not be used to compel adherence to a given method of medical management, which method should be finally determined after taking account of the conditions at the relevant medical institution (staff levels, experience, equipment, etc.), and the characteristics of the individual patient. However, responsibility for the results of treatment rests with those who are directly engaged therein, and not with the consensus group.

## Methods

A computerized search was done by the bibliographer in different databanks (MEDLINE, Scopus, EMBASE). Citations were included for the period between January 1990 and October 2019 using the primary search strategy: liver, injuries, trauma, hepatic, adult, pediatric, hemodynamic instability/stability, angioembolization, management, nonoperative, conservative, operative, surgery, diagnosis, and follow-up, combined with AND/OR. No search restrictions were imposed. The dates were selected to allow comprehensive published abstracts of clinical trials, consensus conference, comparative studies, congresses, guidelines, government publication, multicenter studies, systematic reviews, meta-analysis, large case series, original articles, and randomized controlled trials. Case reports and small case series were excluded. Narrative review articles were also analyzed to determine if other cited studies should be included.

The level of evidence (LE) was evaluated using the GRADE system [7] (Table 1).

**Table 1:** GRADE system to evaluate the level of evidence and recommendation

Grade of recommendation	Clarity of risk/benefit	Quality of supporting evidence	Implications
1A			
Strong recommendation, high-quality evidence	Benefits clearly outweigh risk and burdens, or vice versa	RCTs without important limitations or overwhelming evidence from observational studies	Strong recommendation, applies to most patients in most circumstances without reservation
1B			
Strong recommendation, moderate-quality evidence	Benefits clearly outweigh risk and burdens, or vice versa	RCTs with important limitations (inconsistent results, methodological flaws, indirect analyses, or imprecise conclusions) or exceptionally strong evidence from observational studies	Strong recommendation, applies to most patients in most circumstances without reservation
1C			
Strong recommendation, low-quality or very low-quality evidence	Benefits clearly outweigh risk and burdens, or vice versa	Observational studies or case series	Strong recommendation but subject to change when higher quality evidence becomes available
2A			
Weak recommendation, high-quality evidence	Benefits closely balanced with risks and burden	RCTs without important limitations or overwhelming evidence from observational studies	Weak recommendation, best action may differ depending on the patient, treatment circumstances, or social values
2B			
Weak recommendation, moderate-quality evidence	Benefits closely balanced with risks and burden	RCTs with important limitations (inconsistent results, methodological flaws, indirect, or imprecise) or exceptionally strong evidence from observational studies	Weak recommendation, best action may differ depending on the patient, treatment circumstances, or social values
2C			
Weak recommendation, low-quality or very low-quality evidence	Uncertainty in the estimates of benefits, risks, and burden; benefits, risk, and burden may be closely balanced	Observational studies or case series	Very weak recommendation; alternative treatments may be equally reasonable and merit consideration

A group of experts in the field coordinated by a central coordinator was contacted to express their evidence-based opinion on several issues about the pediatric (< 16 years old) and adult liver trauma [8, 9]. Hepatic trauma was assessed by the anatomy of the injury, type of injury (blunt and penetrating injury), management (conservative and operative management), and type of patient (adults, pediatrics). Through the Delphi process, different issues were discussed in subsequent rounds. The central coordinator assembled the different answers derived from each round. Each version was then revised and improved. An expert group discussed the definitive version. The final version about on agreement was reached resulted in the present manuscript. Statements are summarized in Table 4.

## Definitions

In adult patients, hemodynamic instability is considered the condition in which admission systolic blood pressure is < 90 mmHg with clinical evidence of hemorrhagic shock with skin vasoconstriction (cool, clammy, decreased capillary refill), altered level of consciousness and/or shortness of breath, or > 90 mmHg but requiring bolus infusions/transfusions and/or vasopressor drugs and/or admission base excess (BE) > -5 mmol/l or transfusion requirement of at least > 4 units of packed red blood cells within the first 8 h. Transient responder patients (adult and pediatric) are those showing an initial response to adequate fluid resuscitation, but then subsequent signs of ongoing blood loss and perfusion deficits. These patients have an initial response to therapy but do not reach sufficient stabilization to undergo endovascular procedures or NOM.

In pediatric patients, hemodynamic stability is considered a systolic blood pressure of 70 mmHg plus twice the child’s age in years. An acceptable hemodynamic status in children is considered a positive response to fluid resuscitation: 2 boluses of 20 mL/kg of crystalloid replacement should be administered before blood replacement leading to heart rate reduction, cleared sensorium, return of peripheral pulses, normal skin color, increase in blood pressure and urinary output, and an increase in warmth of the skin in the extremities. Clinical judgment however is fundamental in evaluating pediatric patients.

## WSES classification

The WSES classification (Table 2) divides liver injuries into four classes considering the AAST-OIS classification (Table 3) and the hemodynamic status (Table 4):

- Minor (WSES grade I)
- Moderate (WSES grade II)
- Severe (WSES grade III and IV)

**Table 2:** WSES liver trauma classification

	WSES grade	AAST	Hemodynamic
Minor	WSES grade I	I–II	Stable
Moderate	WSES grade II	III	Stable
Severe	WSES grade III	IV–V	Stable
	WSES grade IV	I–VI	Unstable

**Table 3:**
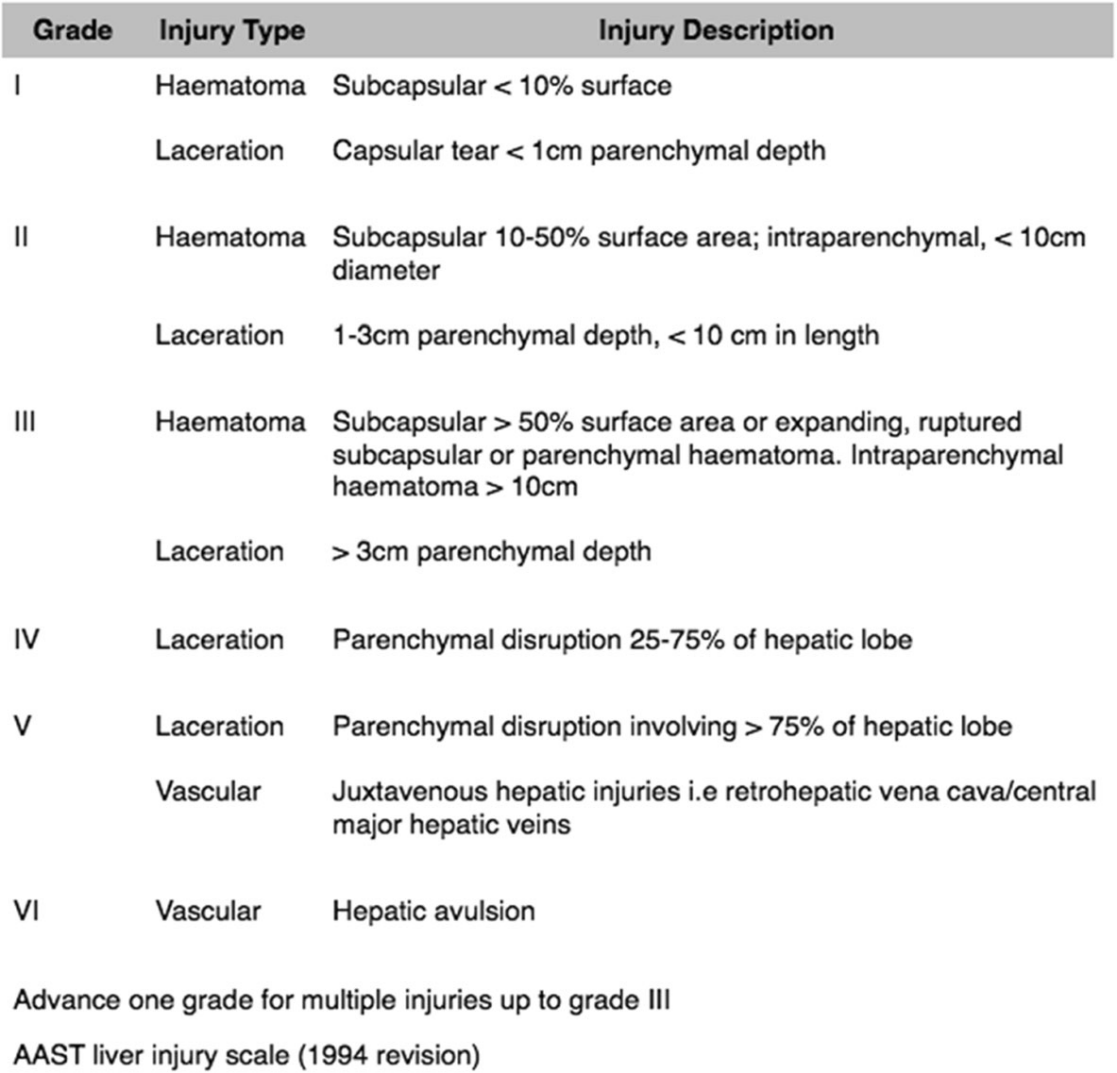
AAST liver trauma classification

**Table 4:** Statements summary

	Statements
Diagnostic procedures	- The diagnostic methods on admission are determined by the hemodynamic status (GoR 1A). - E-FAST is rapid in detecting intra-abdominal free fluid (GoR 1A). - CT scan with intravenous contrast is the gold standard in hemodynamically stable trauma patients (GoR 1A).
Non-operative management (NOM)	- NOM should be the treatment of choice for all hemodynamically stable minor (WSES I) (AAST I–II), moderate (WSES II) (AAST III), and severe (WSES III) (AAST IV–V) injuries in the absence of other internal injuries requiring surgery (GoR 2A). - In patients considered transient responders with moderate (WSES II) (AAST III) and severe (WSES III) (AAST IV–V) injuries, NOM should be considered only in selected settings provided the immediate availability of trained surgeons, operating room, continuous monitoring ideally in an ICU or ER setting, access to angiography, angioembolization, blood and blood products, and in locations where a system exists to quickly transfer such patients to higher level of care facilities (GoR 2B). - A CT scan with intravenous contrast should always be performed in patients being considered for NOM (GoR 2A). - AG/AE may be considered as a first-line intervention in hemodynamically stable patients with arterial blush on CT scan (GoR 2B). - In hemodynamically stable children, the presence of contrast blush on CT scan is not an absolute indication for AG/AE (GoR 2B). - Serial clinical evaluations (physical exams and laboratory testing) must be performed to detect a change in clinical status during NOM (GoR 2A). - NOM should be attempted in the setting of concomitant head trauma and/or spinal cord injuries with reliable clinical exam, unless the patient could not achieve specific hemodynamic goals for the neurotrauma and the instability might be due to intra-abdominal bleeding (GoR 2B). - Intensive care unit admission in isolated liver injury may be required only for moderate (WSES II) (AAST III) and severe (WSES III) (AAST IV–V) lesions (GoR 2B). - In selected cases where an intra-abdominal injury is suspected in the days after the initial trauma, interval laparoscopic exploration may be considered as an extension of NOM and a means to plan patient management in a step-up treatment strategy (GoR 2C). - In low-resource settings, NOM could be considered in patients with hemodynamic stability without evidence of associated injuries, with negative serial physical examinations and negative imaging and blood tests (GoR 2C).
Operative management (OM)	- Hemodynamically unstable and non-responder patients (WSES IV) should undergo OM (GoR 2A). - Primary surgical intention should be to control the hemorrhage and bile leak and initiation of damage control resuscitation as soon as possible (GoR 2A). - Major hepatic resections should be avoided at first and only considered in subsequent operations, in a resectional debridement fashion in cases of large areas of devitalized liver tissue done by experienced surgeons (GoR 2B). - Angioembolization is a useful tool in case of persistent arterial bleeding after non-hemostatic or damage control procedures (GoR 2A). - Resuscitative endovascular balloon occlusion of the aorta (i.e., REBOA) may be used in hemodynamically unstable patients as a bridge to other more definitive procedures for hemorrhage control (GoR 2B).
Short- and long-term follow-up	- Intrahepatic abscesses may be successfully treated with percutaneous drainage (GoR 2A). - Delayed hemorrhage without severe hemodynamic compromise may be managed at first with AG/AE (GoR 2A). - Hepatic artery pseudoaneurysm should be managed with AG/AE to prevent rupture (GoR 2A). - Symptomatic or infected bilomas should be managed with percutaneous drainage (GoR 2A). - Combination of percutaneous drainage and endoscopic techniques may be considered in managing post-traumatic biliary complications not suitable for percutaneous management alone (GoR 2B). - lavage/drainage and endoscopic stenting may be considered as the first approach in delayed post-traumatic biliary fistula without any other indication for laparotomy (GoR 2B). - Laparoscopy as initial approach should be considered in cases of delayed surgery, so as to minimize the invasiveness of surgical intervention and to tailor the procedure to the lesion (GoR 2B).
Thrombo-prophylaxis, feeding, and mobilization	- Mechanical prophylaxis is safe and should be considered in all patients with no absolute contraindication (GoR 2A). - LMWH-based prophylaxis should be started as soon as possible following trauma and may be safe in selected patients with liver injury treated with NOM (GoR 2B). - In those patients taking anticoagulants, individualization of the risk-benefit balance of anticoagulant reversal is suggested (GoR 1C). - Early mobilization should be achieved in stable patients (GoR 2A). - In the absence of contraindications, enteral feeding should be started as soon as possible (GoR 2A).

Minor hepatic injuries:

- WSES grade I includes AAST-OIS grade I–II hemodynamically stable lesions.

Moderate hepatic injuries:

- WSES grade II includes AAST-OIS grade III hemodynamically stable lesions.

Severe hepatic injuries:

- WSES grade III includes AAST-OIS grade IV–V hemodynamically stable lesions.
- WSES grade IV includes AAST-OIS grade I–VI hemodynamically unstable lesions.

**Fig. 1:**
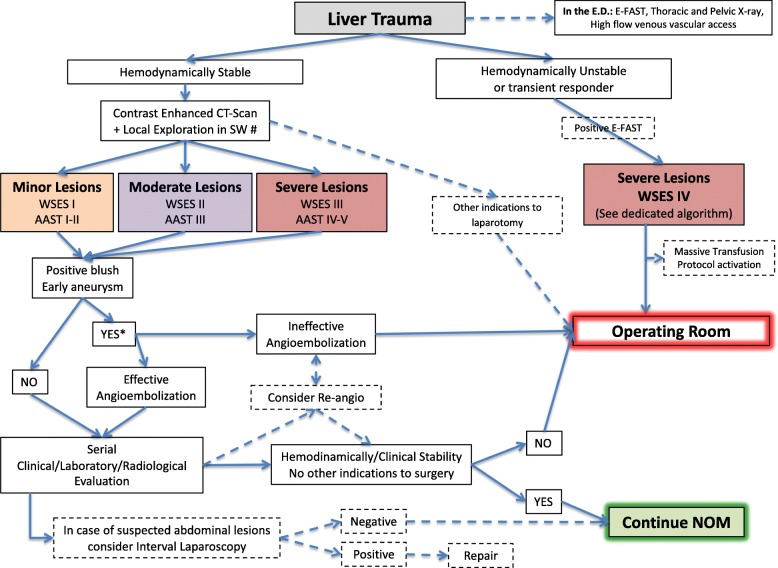
Liver trauma management algorithm (SW: stab wound. Number sign indicates wound exploration near the inferior costal margin should be avoided if not strictly necessary. Asterisk indicates angioembolization should be always considered for adults, only in selected patients and in selected centers for pediatrics)

**Fig. 2:**
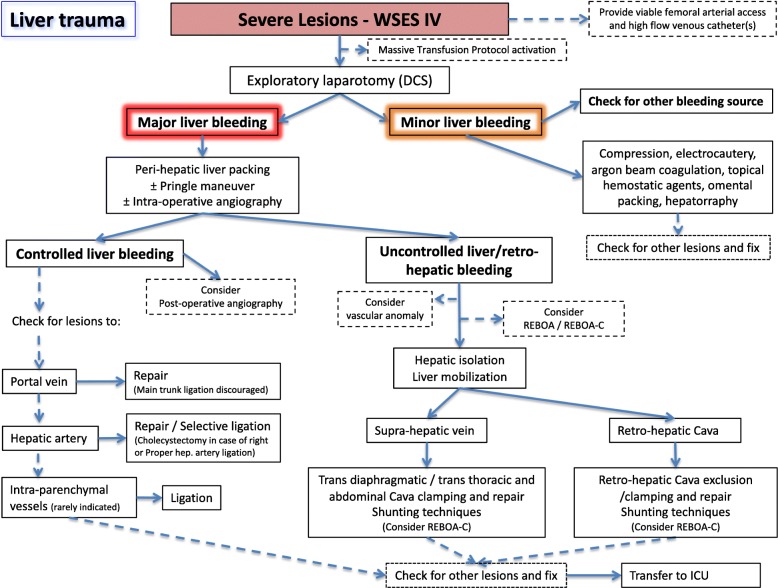
Hemodynamically unstable liver trauma management algorithm (DCS: damage control surgery, ICU: intensive care unit, REBOA-C: REBOA-cava)

Based on the present classification, we suggest two management algorithms: one general (Fig. 1) and one specifically dedicated to hemodynamically unstable patients (Fig. 2).

### Diagnosis

- The diagnostic methods on admission are determined by the hemodynamic status (GoR 1A).
- Extended-focused abdominal sonography for trauma (E-FAST) is rapid in detecting intra-abdominal free fluid (GoR 1A).
- CT scan with intravenous contrast is the gold standard in hemodynamically stable trauma patients (GoR 1A).

Careful physical examination is of paramount importance in determining the need for exploratory laparotomy [10]. E-FAST is useful and generally reliable in trauma in general. However, abdominal ultrasound may be falsely negative due to clotted blood or suboptimal quality views [11–13]. In the pediatric population, reported sensitivity and specificity ranges from 42 to 52% and 96 to 98%, with a negative predicting value for intra-abdominal fluid of 93–96% [8, 9, 14–16]. The low sensitivity of E-FAST in hemodynamically stable pediatric patients may warrant further investigation, specifically contrast-enhanced ultrasound (US) or abdomen/pelvis CT scan or magnetic resonance, in hemodynamically stable pediatric patients with a high degree of suspicion for intra-abdominal injury (abnormal physical examination, abnormal laboratory values, or other radiologic studies).

Computed tomography (CT) scan is considered the gold standard in trauma imaging assessment with a sensitivity and specificity approaching 96–100% [17–19]. CT must be immediately available and performed only in hemodynamically stable or stabilized patients or in those who transiently responded to fluid resuscitation in special circumstances and under the supervision of the trauma team [20, 21]. Delayed-phase CT helps in differentiating patients with active bleeding from those with contained vascular injuries [22]. This data is important to reduce the risk of discrepancy between CT scan images and angiographic images (only 47% of patients have a confirmation of the CT findings at angiography) [22]. Active contrast extravasation is a sign of active hemorrhage [23]. CT scan may help in subsequent surgical procedures and angiography/angioembolization (AG/AE) [24–32].

Diagnostic peritoneal lavage (DPL) should be considered diagnostic modality in low-resource settings, where CT scan or US is not promptly available [33]. It should be considered in the presence of massive subcutaneous emphysema in a shocked patient in whom ultrasound cannot be done and/or in the presence of free peritoneal fluid without solid organ injury in a hemodynamically stable patient. The possibility of DPL-related complications (up to 2%) should be considered [33].

### Non-operative management

- NOM should be the treatment of choice for all hemodynamically stable minor (WSES I) (AAST I–II), moderate (WSES II) (AAST III), and severe (WSES III) (AAST IV–V) injuries in the absence of other internal injuries requiring surgery (GoR 2A).
- In patients considered transient responders with moderate (WSES II) (AAST III) and severe (WSES III) (AAST IV–V) injuries, NOM should be considered only in selected settings provided the immediate availability of trained surgeons, operating room, continuous monitoring ideally in an ICU or ER setting, access to angiography, angioembolization, blood, and blood products, and in locations where a system exists to quickly transfer such patients to higher level of care facilities (GoR 2B).
- A CT scan with intravenous contrast should always be performed in patients being considered for NOM (GoR 2A).
- AG/AE may be considered as a first-line intervention in hemodynamically stable patients with arterial blush on CT scan (GoR 2B).
- In hemodynamically stable children, the presence of contrast blush on CT scan is not an absolute indication for AG/AE (GoR 2B).
- Serial clinical evaluations (physical exams and laboratory testing) must be performed to detect a change in clinical status during NOM (GoR 2A).
- NOM should be attempted in the setting of concomitant head trauma and/or spinal cord injuries with reliable clinical exam, unless the patient could not achieve specific hemodynamic goals for the neurotrauma and the instability might be due to intra-abdominal bleeding (GoR 2B).
- Intensive care unit admission in isolated liver injury may be required only for moderate (WSES II) (AAST III) and severe (WSES III) (AAST IV–V) lesions (GoR 2B).
- In selected cases where an intra-abdominal injury is suspected in the days after the initial trauma, interval laparoscopic exploration may be considered as an extension of NOM and a means to plan patient management in a step-up treatment strategy (GoR 2C).
- In low-resource settings, NOM could be considered in patients with hemodynamic stability without evidence of associated injuries, with negative serial physical examinations and negative imaging and blood tests (GoR 2C).

Absolute requirements for NOM are hemodynamic stability and absence of other lesions requiring surgery [9, 15, 34–39]. In hemodynamically stable patients without other associated injuries requiring OM, NOM is considered the standard of care [8, 14, 15]. The concept is valid for both: blunt (BT) and penetrating trauma (PT). Attempting NOM in moderate (WSES II) (AAST-OIS III) and severe (WSES III) (AAST-OIS IV–V) blunt or penetrating injuries requires the ability to diagnose all associated injuries and to provide intensive management (continuous clinical monitoring, serial hemoglobin monitoring, and around-the-clock availability of trained surgeons, CT scanning, angiography, OR, and blood and blood products) [16, 40–44].

As a general consideration, great attention should be paid in selecting PT for NOM especially in the case of gunshot wound (GSW) and even more if thoraco-abdominal. They should be considered for NOM only in centers with experience in dealing with PT. Even in patients presenting with stable conditions and with no evidence of other intra-abdominal/internal injuries, interval laparoscopy should be always considered in order to confirm the absence of other injuries requiring surgical repair.

In PT, NOM feasibility has been reported [35–37, 45–49] with 50% and 85% success rate of NOM for stab wounds (SW) in anterior and posterior abdomen respectively [34, 50]. Similar managing strategy can be applied to GSWs [35, 45]. Necessary distinction between low- and high-energy penetrating trauma however is mandatory when deciding for OM or NOM. Low-energy PT (SW and low-energy GSW) may be safely treated with NOM at first, provided the patient is hemodynamically stable and no other injuries require surgery. In considering NOM, interval laparoscopy should be considered to rule out missed intra-abdominal injuries. High-energy GSW and other ballistic injuries are less amenable to NOM, and in 90% of cases, OM is required [34, 36, 51]. In abdominal GSWs, up to 25% of non-therapeutic laparotomy has been reported [51], confirming the need to have strict selection criteria for OM or NOM even in the GSW cohort. Associated head and spinal cord injuries (that preclude affordable clinical examination) and significant reduction in hemoglobin requiring > 4 units of blood transfusion in the first 8 h [34, 45] have been suggested as predictive criteria of NOM failure in abdominal GSWs.

Patient selection is influenced by the diagnostic capability and accuracy. In fact, the accuracy of CT scan in SWs has been questioned [37, 50]. Even in the presence of a negative CT scan, exploratory laparoscopy/laparotomy may be necessary [37]. Interval laparoscopy is a useful tool to be considered in obese patients or in the presence a long and tangential wound tract or when the trajectory is difficult to determine on CT scan [34, 37]. In anterior abdominal SW, local wound exploration (LWE) is generally accurate in evaluating penetration depth; small external wounds may be enlarged for precise LWE and determination of anterior fascia violation [34, 35]. LWE, however, may be misleading, and patients should be admitted for observation if equivocal. Wounds close to the inferior costal margin should be evaluated by LWE with caution and only if strictly necessary.

GSWs undergoing NOM may warrant a CT scan to determine the trajectory [45, 51]. CT scan specificity and sensitivity of 96% and 90.5% respectively for GSWs requiring laparotomy have been reported [52]. The gold standard to decide for OM or NOM remains the clinical examination [34, 51] associated with laboratory and radiological evaluation. Strict clinical and hemoglobin evaluation should be done (every 6 h for at least 24 h); after index CT scan allowing for NOM, serial ecoghraphical evaluation may be utilized to help in defining patient clinical evolution. Once stabilized, patients are usually transferred from ICU to the ward [35, 45, 50].

NOM is contraindicated if free intra- or retro-peritoneal air, free intra-peritoneal fluid in the absence of solid organ injury, localized bowel wall thickening, bullet tract close to hollow viscus with surrounding hematoma [46], and in high-energy penetrating trauma are detected at CT scan.

In selected centers, AE is considered as an “extension” of NOM in patients with liver injuries presenting with ongoing resuscitative needs [9, 53, 54]. If required, AE can be safely repeated.

In children, the use of primary hepatic AE has been reported rarely and is debated even in the presence of arterial blush where it seems to increase NOM failure rates [55], or according to some studies, it does not correlate with decrease odds of laparotomy [30]. In the pediatric population, AE use is associated with older age and is not completely defined in terms of efficacy and cost-effectiveness, especially in low-resource settings [30, 55–61]. Some authors, however, identify the presence of active contrast extravasation as an independent predictor for pseudoaneurysm (PSA) formation in children, regardless of injury grade. This suggests a thorough follow-up during NOM of these patients, so to obtain an early identification and angiographic treatment of PSA [62].

The biggest risk of NOM in penetrating trauma is a missed abdominal injury, especially hollow viscus perforation [34, 46]. However, no increase in mortality rates with missed hollow viscus perforation has been reported in patients without peritonitis on admission [63]. As a counterpart, non-therapeutic laparotomy leads to an increase in morbidity [63]. Moreover, OM in penetrating liver injuries has a higher liver-related complication rate (50–52%) compared to blunt injuries [34, 46].

During NOM for liver injuries, no standard early follow-up and monitoring protocols exist in adult or in children [34]. Serial clinical evaluation and hemoglobin measurement represent the cornerstone in evaluating NOM patients [14]. Bedsides, US may represent an affordable tool during early follow-up. Presence of large subcapsular hematomas is not a strict indication for OM, but a higher risk of NOM failure exists. In any case, these patients should undergo serial blood test: increasing levels of transaminases could indicate the presence of intrahepatic parenchymal ischemia or rare cases of torsion of suprahepatic veins [64]. ICU admission may be indicated for moderate (WSES II) (AAST III) and severe (WSES III–IV) (AAST IV–V) liver trauma in order to reduce the mortality risk [26].

If available, interval laparoscopy during NOM provides important information about the evolution of the injury. Laparoscopy should be considered an important tool in the NOM of liver injuries, and it could be used as a bridge strategy to plan an immediate or subsequent laparoscopic/laparotomy intervention [65].

Particular attention should be paid in managing hemodynamically stable patients with liver trauma associated with spinal trauma (ST) and severe traumatic brain injury (STBI). In blunt trauma, NOM should apply to all patients with no other indication to laparotomy. However, the optimal management of concomitant STBI and/or ST and penetrating liver injuries is debated and OM in general could be suggested as safer [45, 48, 66].

Patients affected by neurotrauma (i.e., spinal cord or moderate-severe traumatic brain injury) in fact, for several instances, differ from the others because they need a higher perfusion pressure to adequately supply oxygen to the brain and to the spinal cord to reduce the subsequent burden of disability and mortality. A disruption of the normal blood flow regulation in the central nervous system (CNS) characterizes the trauma and eventually leads to a blood flow dependent on perfusion pressure in ischemic tissue [67]. Specific hemodynamic goals for ST and STBI are defined as SBP > 110 mmHg and/or a CPP between 60 and 70 mmHg in the case of moderate/severe TBI and an MBP > 80 mmHg in case of ST [68, 69]. To date, no study specifically addressed the NOM of abdominal solid organ injuries in the neurotrauma patient, and several authors have considered it an exclusion criterion from NOM [45, 48, 70]. However, since the first goal is to have a stable patient with adequate perfusion pressure, there is no rationale in denying NOM to these patients, as long as the specific hemodynamic goals are met.

### Operative management

- Hemodynamically unstable and non-responder patients (WSES IV) should undergo OM (GoR 2A).
- Primary surgical intention should be to control the hemorrhage and bile leak and initiation of damage control resuscitation as soon as possible (GoR 2A).
- Major hepatic resections should be avoided at first and only considered in subsequent operations, in a resectional debridement fashion in cases of large areas of devitalized liver tissue done by experienced surgeons (GoR 2B).
- Angioembolization is a useful tool in case of persistent arterial bleeding after non-hemostatic or damage control procedures (GoR 2A).
- Resuscitative endovascular balloon occlusion of the aorta (i.e., REBOA) may be used in hemodynamically unstable patients as a bridge to other more definitive procedures for hemorrhage control (GoR 2B).

At laparotomy, if no major bleeding is present, compression alone or electrocautery, bipolar devices, argon beam coagulation, topical hemostatic agents, simple suture of the hepatic parenchyma, or omental patching may be sufficient to stop the bleeding [34, 66, 71–73].

In case of major hemorrhage, more aggressive procedures including manual compression and hepatic packing, ligation of vessels in the wound, hepatic debridement and finger fracture, balloon tamponade, shunting procedures, or hepatic vascular isolation and exclusion may be used [64, 74]. Of paramount importance is to provide simultaneous intraoperative intensive resuscitation with early institution of a massive transfusion protocol (MTP) aiming to maintain organ perfusion and ultimately reverse all trauma-induced physiological derangements [34, 71, 73, 75].

In case of evident injury to the proper hepatic artery, an attempt to control and repair it should be made. If not effective or not possible, selective hepatic artery ligation should be considered as a viable option. If the injury is on the right or left branches of the proper hepatic artery, selective ligation is advisable. If the right or common hepatic artery must be ligated, cholecystectomy should be performed to avoid gallbladder necrosis [2, 76]. If the patient’s condition allows for it, post-operative AE represents a viable alternative allowing hemorrhage control while reducing complications [34, 66, 71, 77]. Hepatic artery ligation increases the risk of hepatic necrosis, abscesses, and biloma formation [34].

Portal vein injuries should be repaired primarily. Portal vein main branch ligation should not be considered and should be avoided because of the high risk of liver necrosis or massive bowel edema. If no other option exists, ligation can be used, but only in patients with an intact hepatic artery. Liver packing or liver resection should be preferred to ligation in case of lobar or segmental/subsegmental portal venous branch injuries [34, 76].

Whenever Pringle maneuver or arterial control fails and bleeding persists, the presence of an aberrant hepatic artery should be considered. If the bleeding comes from behind the liver, retro-hepatic caval or hepatic vein injury should be highly suspected [34, 77]. Three viable options exist for the management of retrohepatic caval/suprahepatic venous injuries: (1) tamponade with hepatic packing, (2) direct repair (with or without vascular isolation), and (3) lobar resection [38, 78–80]. Liver packing is the least risky method to temporarily deal with severe venous injuries [34, 66, 81–83]. Direct venous repair is difficult especially in non-experienced hands, with high mortality rates [34, 66].

Different techniques of hepatic vascular exclusion with shunting procedures have been described, most of them anecdotally. The veno-veno bypass (femoral vein and inferior mesenteric vein to axillary or jugular vein by pass) and the use of fenestrated stent grafts are the most frequently used [66, 71, 76, 84]. The atrio-caval shunt bypasses the retro-hepatic cava blood through the right atrium using a chest tube put into the inferior vena cava. Mortality rates in such a complicated situations are very high and usually related to the fact that the decision to perform the shunt is made late in the case [71]. Complete vascular exclusion of the liver is generally poorly tolerated in the unstable patient with major blood loss [34].

Resuscitative endovascular balloon occlusion of the aorta (REBOA) catheter in zone I should be considered if despite all damage control procedures, there is still active surgical bleeding. Simultaneously, the large high flow femoral venous catheter should be exchanged over a guide wire to an introducer with the aim of floating up and inflating a resuscitative endovascular balloon occlusion of the vena cava (REBOVC) at the level of the retro-hepatic vena cava. The goal is to achieve proximal and distal vascular control of a possible retro-hepatic/supra-hepatic vessel injury with the REBOVC and ultimately obtaining complete combined endovascular/open liver isolation with the Pringle maneuver. A supra-diaphragmatic central venous access must be obtained prior to inflating the REBOA/REBOVC [85–91].

In cases of liver avulsion or total crush injury, when a total hepatic resection is indicated, hepatic transplantation has been described [76]. A retrospective study based on the European Liver Transplant Registry identifies an ISS score less than 33 for recipient selection, so to avoid futile procedures [92].

Anatomic hepatic resection may seldom be considered as a surgical option [6, 93, 94]. In unstable patients and during damage control surgery, it should be avoided, but in case of need, a non-anatomic resection is safer and easier [34, 66, 71, 76]. For staged liver procedures, either anatomic or non-anatomic resections may be safely performed by experienced surgeons [76].

Temporary abdominal closure may be indicated if the risk of abdominal compartment syndrome is high or in those situation where a “second look” operation is needed [71–73].

Two principal indications for post-operative angiography-embolization (AG-AE) have been proposed: (1) after initial operative hemostasis, in stable or stabilized patients with contrast blush at completion CT scan; and (2) as adjunctive hemostatic tool in patients with uncontrolled suspected arterial bleeding despite emergency laparotomy and hemostasis attempt [34, 54, 95–99]. Recent evidence suggests that routine use of immediate post-damage control hepatic angiography reduces mortality in grade IV/V hepatic injuries [100].

### Complications

- Intrahepatic abscesses may be successfully treated with percutaneous drainage (GoR 2A).
- Delayed hemorrhage without severe hemodynamic compromise may be managed at first with AG/AE (GoR 2A).
- Hepatic artery pseudoaneurysm should be managed with AG/AE to prevent rupture (GoR 2A).
- Symptomatic or infected bilomas should be managed with percutaneous drainage (GoR 2A).
- Combination of percutaneous drainage and endoscopic techniques may be considered in managing post-traumatic biliary complications not suitable for percutaneous management alone (GoR 2B).
- Laparoscopic lavage/drainage and endoscopic stenting may be considered as the first approach in delayed post-traumatic biliary fistula without any other indication for laparotomy (GoR 2B).
- Laparoscopy as initial approach should be considered in cases of delayed surgery, so as to minimize the invasiveness of surgical intervention and to tailor the procedure to the lesion (GoR 2B).

In blunt hepatic trauma, particularly after high-grade injury, complications occur in 12–14% of patients [9, 66]. Diagnostic tools for complications after NOM include clinical examination, blood tests, ultrasound, and CT scan. Routine follow-up with CT scan is not necessary unless there is clinical suspicion of a complication [6, 9, 66]. In the presence of abnormal inflammatory response, abdominal pain, fever, jaundice, or drop of hemoglobin level, repeated CT scan is recommended [9]. Bleeding, abdominal compartment syndrome, infections (abscesses and other infections), biliary complications (bile leak, hemobilia, biloma, biliary peritonitis, biliary fistula), and liver necrosis are the most frequent complications associated with NOM [16, 66]. Ultrasound is useful in the assessment of bile leak/biloma in grade IV–V injuries, especially with a central laceration.

Re-bleeding or secondary hemorrhage is the most frequently reported complications after NOM as in subcapsular hematoma or pseudo-aneurysm (PSA) rupture (range 1.7–5.9%) with a mortality rate up to 18% [9, 66, 101, 102]. In the majority of cases (69%), “late” bleeding can be treated non-operatively [9, 66].

Hepatic artery PSA is a rare complication with a prevalence of 1% [103]. Asymptomatic PSA should be treated as early as possible with AE because of the high risk of rupture and the associated high morbidity [34, 104, 105]. In patients with melena or hematemesis following liver trauma, bleeding from the ampulla of Vater (hemobilia) is highly suggestive of ruptured intrahepatic PSA [106, 107]. AE is the treatment of choice [6, 34, 66]. In the presence of intrahepatic bilio-venous fistula (frequently associated with bilemia), endoscopic retrograde cholangiopancreatography (ERCP) represents an effective tool [108].

Biliary complications include biloma, biliary fistula, bilhemia, and bile peritonitis (incidence 2.8–30%) [8, 40]. Most traumatic bilomas regress spontaneously. Enlarging, symptomatic or infected bilomas can be successfully managed with percutaneous drainage. Percutaneous drainage may be combined with therapeutic ERCP with eventual endobiliary stent placement [9, 101, 109–111]. Bile peritonitis has been usually treated with laparotomy. Combination of laparoscopic irrigation/drainage and endoscopic bile duct stent placement may represent a valid alternative [101, 102, 112, 113].

Abscesses are rare after NOM and usually happen in severe lesions (prevalence 0.6–7%) [9, 66, 114–117]. CT scan or ultrasound-guided percutaneous drainage is the treatment of choice with high success rate and no reported mortality [106]. In the presence of necrosis and devascularization of hepatic segments, surgical management may be indicated whenever affecting patient condition [34, 66].

Generally, once stabilization of traumatized patient is obtained, late complications should be managed preferentially by minimally invasive procedures. Laparoscopy and endoscopy are part of this approach, which became possible in a delayed surgery setting [64, 65, 118, 119].

### Thromboprophylaxis, feeding, and mobilization

- Mechanical prophylaxis is safe and should be considered in all patients with no absolute contraindication (GoR 2A).
- LMWH-based prophylaxis should be started as soon as possible following trauma and may be safe in selected patients with liver injury treated with NOM (GoR 2B).
- In those patients taking anticoagulants, individualization of the risk-benefit balance of anticoagulant reversal is suggested (GoR 1C).
- Early mobilization should be achieved in stable patients (GoR 2A).
- In the absence of contraindications, enteral feeding should be started as soon as possible (GoR 2A).

Venous thromboembolism (VTE) is one of the great risks of trauma victims, because patients enter a hyper-coagulation state within 48 h from injury [120–122]. More than 50% of patients without thrombo-prophylaxis may develop deep vein thrombosis (DVT) and subsequent pulmonary embolism (PE) which carries a morality rate up to 50% [120, 121]. PE is the third leading cause of death in trauma patients.

No differences in complication, mortality, and NOM failure rate were demonstrated when thrombo-prophylaxis was administered within and after 48 and 72 h from the initial injury in patients without STBI and BST [123–125]. Early mobilization is not related to NOM failure and secondary bleeding [126]. However, VTE rates seem to be over fourfold when LMWH is administered > 72 h from admission [120].

In patients taking anticoagulants, it is important to evaluate the eventual need for reversal therapy in order to balance the risk of bleeding against the benefit of preventing thrombotic complications. Poor outcomes derive from the failure to restore the anticoagulation as soon as possible [127].

Early enteral feeding is associated with improved clinical outcomes when administered within the first 72 h from admission in ICU [128], and it should be delayed only in cases of uncontrolled shock, use of vasopressor therapy, uncontrolled hypoxaemia and acidosis, uncontrolled upper GI bleeding, gastric aspirate > 500 ml/6 h, bowel ischemia, bowel obstruction, abdominal compartment syndrome, and high-output fistula without distal feeding access [129]. Oral intake, when possible, should be initiated after 24–48 h from the traumatic event.

### Follow-up

Mandatory late follow-up imaging is not indicated, and it should be used only if the patient’s clinical condition and/or symptoms indicating a complication require it for diagnosis. The majority of liver lesions heal in about 4 months [14, 66]. After moderate and severe liver injuries, patients may usually resume normal physical activities after 3–4 months.

During the recovery phase, patients should be encouraged to not remain alone for long periods and to return immediately to the hospital in case of increasing abdominal pain, lightheadedness, nausea, or vomiting [14, 34].

## Conclusions

Management of liver trauma is multidisciplinary. When feasible, non-operative management should always be considered as the first option in adult and in the pediatric populations. For this reason, clinical condition, anatomical injury grade, and associated injuries should be considered together in deciding the best treatment option.

## Data Availability

Not applicable

## Abbreviations

NOM: Non-operative management
OM: Operative management
AAST: American Association for Surgery for Trauma
WSES: World Society of Emergency Surgery
PTS: Panamerican Trauma Society
ATLS: Advanced trauma life support
ERCP: Endoscopic retrograde cholangiopancreatography
BLT: Blunt liver trauma
SW: Stab wounds
GSW: Gunshot wound
DCS: Damage control surgery
OR: Operating room
AG: Angiography
AE: Angioembolization
EVTM: Endovascular bleeding and trauma management
STBI: Severe traumatic brain injury
ST: Spine trauma
CNS: Central nervous system
PSA: Pseudoaneurysm
REBOA: Resuscitative endovascular balloon occlusion of the aorta
MTP: Massive transfusion protocol
